# The Prevalence of Pneumonia in Chicago, in 1882; Its Chief Causes and Characteristics

**Published:** 1883-05

**Authors:** N. S. Davis


CHICAGO MEDICAL

JOURNAL & EXAMINER.

VOL. XLVI.—MAY, 1883.—NO. 5.

### Original Communications.

### ARTICLE I.

THE PREVALENCE OF PNEUMONIA IN CHICAGO IN 1882; ITS CHIEF CAUSES AND CHARACTERISTICS. By N. S. DAVIS, M.D., etc. (Read to the Chicago Medical Society, March 19, 1883.)

That acute pneumonia, pneumonitis, pneumonic fever, lung fever, and winter fever, as it is variously called by different parties, is one of the most common, as well as most fatal, forms of disease the physician has to encounter, is shown by the mortuary statistics of all civilized countries. A more definite and correct idea of the prevalence of pneumonitis, or acute inflammation of the parenchyma of the lungs, and the mortality arising therefrom, is conveyed by the following statistics, taken from the official records of mortality in cities representing the different parts of our country, for the year 1882: The whole number of deaths from pneumonia reported to the health office in this city during the year just named, is 782; from broncho-pneumonia, 42, and from pleuro-pneumonia, 20.

As bronchitis and pleurisy are much less dangerous to life than pneumonia, it is proper to attribute the deaths reported under the complications named to the latter disease. If so, the aggregate of deaths from pneumonia in this city during the year 1882, was 844; or one in every 597 of the population, as given in the census of 1880. In the same year, the whole number of deaths from pneumonia in Boston was 681, or one in every 532 of the population, as given by the census of 1880. The number of deaths from pneumonia in San Francisco for the year ending June 30, 1882, was 527; or one in every 441 of the population.

The number of deaths reported from pneumonia for the year 1882 in New Orleans, was only 203; or one to every 1,088 of the population.

The four cities I have named represent the northern Atlantic coast, the northern part of the great interior valley of the continent, the southern part of the same valley, and the Pacific coast.

In all of them, the ratio of mortality is sufficient to indicate that the disease is one of the most important with which the physician has to deal. In examining the mortuary tables of previous years, I find the ratio of deaths from the disease in question in Boston and New Orleans for 1882 to be not above the ratio for the two preceding years. In Chicago it appears moderately above the average, as shown by the fact that in 1880 the number returned was 605; in 1881 it was 707, and in 1882 it was 844; the latter being an increase greater than could be explained by any increase of population. In San Francisco, however, the number returned for 1882 is so much in excess of previous years, as to indicate the operation of some unusual cause or causes. For instance, the number of deaths from pneumonia for the five preceding years were: for the year ending June 30, 1877, 306; 1878, 314; 1879, 334; 1880, 368; and 1881, only 296, while, as previously stated, the number for 1882 was 527. If we take the average annual mortality for the five years preceding 1882, it would be 324, or one in every 723 of the population, instead of one for every 441, as in 1882. Again, if, instead of taking the unusually high figures for 1882 in Chicago, we take the average for the three years 1880-1-2, we shall have an annual mortality of 718, or one in every 702 of the population, instead of one for every 597, as given for 1882 alone.

With these corrections, the relative rates of mortality from pneumonia in the four cities named would stand thus: For Boston, one death in every 532 of the population; Chicago, one death in every 702 of the population; San Francisco, one death in every 723 of the population; New Orleans, one death in every 1,088 of the population.

Assuming these figures to be substantially correct, and that the cities chosen fairly represent the northeastern, northern interior, western and southern divisions of the United States, they show, first, that pneumonia is most prevalent and fatal in those sections of country in which the meteorological conditions are most changeable, with a predominance of cold, moisture, and high winds, with a wide range of temperature between the warmest days of summer and the coldest of winter; thereby following the same law in relation to climatic influences as bronchitis, and inflammations of the respiratory passages generally; second, that some of the statements in our standard works on practical medicine need revision and material modification. For example, Dr. A. B. Palmer, in the most recent work on practical medicine issued from the press, says: "Its prevalence is extensive over the globe, and it is found *nearly alike in all latitudes.* Its geographical distribution is different from that of catarrh or bronchitis." (See Science and Practice of Medicine, Vol. II, p. 204.) Dr. Bartholow says: It is a very common disease; it occurs in all degrees of latitude, under every variety of climate, and at all ages." (See Practice of Medicine, p. 325.) Dr. Flint, in his well-known work on the Practice of Medicine, says: "In this country, the disease occurs in the Middle and Southern, much oftener than in the Northern States." (See Practice of Medicine, fifth edition, 1881, p. 168.) Some of the expressions of Dr. Drake, in his work on the Topography and Diseases of the Interior Valley of the Continent, are of the same import as that just quoted from Dr. Flint. And yet the statistics I have quoted from the most reliable records, show not only a wide difference in the ratio of prevalence in different latitudes, but the necessity for directly reversing the statements of Drs. Flint and Drake; the ratio of deaths from the disease being double in Boston, and one-third greater in Chicago, than in New Orleans, Mobile, Augusta, Jacksonville and Charleston ; the two first representing the Northern and Northeastern States, and the five last named the Southern States. The ratio in San Francisco, while showing a degree of prevalence intermediate between the northern and southern climatic belts east of the Rocky mountains, is still more strikingly at variance with statements found in works of high authority.

Thus, Dr. Wilson Fox, in the chapter on pneumonia in Reynolds' System of Medicine, when discussing the influence of climate, says: "Oregon and California appear to enjoy a singular immunity from the disease." (See Reynolds' System of Mediicine, Am. edition, vol. II, page 154.) That the meteorological conditions which constitute the elements of climate, exert much influence over the prevalence of pneumonia, is strongly corroborated by the fact that it is much more prevalent in some seasons of the year than in others. As a rule two thirds of all the cases and deaths occur during the months of December, January, February, March and April. Of the same import, also, is the fact that nearly twice as many males die from the disease as females, caused, doubtless, by their much greater exposure to the sudden and severe meteorological changes during their outdoor occupations. It was not my intention, however, to call your attention so much this evening to the general causes influencing the prevalence of pneumonia, as to some of the more local, and perhaps personal conditions and customs that may exert an important influence both on the number of attacks and on the special characteristics of the disease in particular seasons and localities.

It would be an interesting and profitable item of study to compare closely the different degrees of prevalence of the disease in different years, and in different parts of each year, with the coincident differences in the meteorological conditions, and the materials for such comparisons are now accumulating in the hands of a committee of the American Medical Association, but are not yet ready for full examination.

For instance, there must be some definite reason why pneumonia destroyed 844 lives in this city in 1882, and only 605 in 1880; and equally so, why in 1882 the highest monthly mortality (122) was in January, and the highest in 1880 (i. e., 112), was in April, while in January of that year the number was only 48. The actual causes of such striking variations in the monthly and annual prevalence of an important disease in the same population and locality can and should be ascertained by patient and co-operative observations and records on the part of the members of this society. But in addition to the meteorological or climatic and seasonal influences, there are manifestly others of much etiological importance. For instance, in San Francisco, during the unusual prevalence of pneumonia in 1882, the ratio of deaths from that disease in the 212,520 Caucassian, or white inhabitants, was 1 in 498; in the 22,000 Mongolians, it was 1 in 232, or more than double that among the whites. So in New Orleans during the same year the ratio of deaths was nearly three times as great among the colored as in the white population. In this city and in Boston I have not the official figures for the several races comprising the populations, but I have examined the details sufficiently to know that I am quite within the limits of fact when I say that the mortality from the disease under consideration is thirty-three per cent. greater among the population of foreign birth than among the American part. Closer examination however, shows that these striking differences are not so much due to the inherent susceptibilities of different races, as to modes of living, habits of life and occupations. The aggregation of the Mongolians of San Francisco into the unsanitary Chinese quarters, and the poorly protected, often crowded and unsanitary dwellings of the colored population, not only in New Orleans, but on many plantations and other rural districts, often made worse by dissipation and exposure, afford abundant reasons why they should suffer severely from any acute disease of an endemic character.

It is also a fact that the great mass of foreign population in this and other northern cities belong to the laboring classes, and live in small houses with very small, poorly ventilated sleeping rooms, to which is often added location on damp soil, and from the confined, damp and warm air of these rooms the men go out in the morning to work and abrupt exposure to cold, with vasomotors depressed and blood imperfectly depurated. It requires but a moderate degree of observation to see why far the larger number of the deaths from pneumonia should occur among persons thus situated whether foreign born or native. Another agent which also exerts much influence in favoring the development of pneumonia is the habitual use of alcoholic drinks. Whether the evil effects are owing directly to the presence of the alcohol in the system diminishing nerve sensibility and retarding molecular changes, or indirectly to the greater exposures which drinkers generally encounter, I will not stop now to inquire. The fact that those who indulge in such habits are more liable to attacks, and more liable to die when attacked, is easily verified by observation, and is acknowledged by our best writers.

In regard to the special characteristics of the cases of pneumonia which have occurred in this city the past year, I am not, perhaps, as well able to judge as many of you, my opportunities for observation having been limited mostly to hospital and consultation cases. Such observations as I have made have led me to think the great majority of cases were accompanied by the dullness of expression, softness of pulse, mental wandering, dark color of the bloody sputa and occasional looseness of the bowels, that would require classing them as typhoid in their grade and tendencies.

In some of the cases coming under my observation, the cerebral symptoms were unusually prominent and in two or three cases they were manifest in an unusual manner. The first symptoms were very severe pain in head, most severe in the occipital region, with great restlessness and anxiety, hurried breathing and only little elevation of temperature. After about twenty-four hours the pain drifted to the lower part of one side of the chest, extremely acute, causing the respiration to be short or stifled, very frequent, and pulse sharp and quick ; but the closest examination detected neither the friction of the first stage of pleurisy, nor the crepitant râle of pneumonia ; nor the dullness on percussion of the second stage of either. After the pain in the side and other symptoms mentioned, with temporary feeling of sinking, had continued for nearly forty-eight hours, the pains ceased, the mind became calm, but the pulse and respiration continued short and frequent, like one weary from physical exertion, and giving exaggerated or puerile respiratory murmer, but no râles or dullness over any part of the chest, and no expectoration. During the next twenty-four hours, however, the patient became gradually more dull or drowsy, the respirations shorter, with first crepitant râle over the right side of the chest, which gave place in less than eight hours to submucous râle, some bloody expectoration and marked dullness on percussion, with a weak and frequent pulse. In less than twenty-four hours after the first indications of pneumonic exudation, the whole of the right, and the lower part of the left, lung were completely filled with the exudative material, and the patient died.

Another of this class was marked by a decidedly hysterical order of nervous symptoms; and after suffering excruciating pain, vacillating from the lower half of the left side of the chest to the head, often, for several days without developing any physical signs of either pulmonary or cardiac disease, there supervened well marked symptoms of pneumonia, limited to the lower part of the left lung, quickly followed by endo-carditis. These symptoms had progressed only about twenty-four hours when the patient was seized suddenly with some convulsive movements, and shrieking, as if from intense pain. In this emergency a physician was called in, who administered morphine, both by the mouth and hypodermically. The patient soon fell into a sleep, from which she could be partially aroused six or eight hours later, but lapsed into stupor again, and died about twelve hours after the convulsion. In several other cases the cerebral symptoms came early, and presented the delirium analogous to that often present in the more active grade of typhoid fever. During its continuance, the respiratory movements become less and less efficient; the moist râles more prominent; the pulse soft, weak and frequent; the extremities cool, and skin generally relaxed and wet with perspiration.

In all these cases, the urine was scanty, and deficient in the chlorides, and was sometimes voided with difficulty. One of these patents died at the end of the first week after the attack, another on the eleventh day, aud the rest recovered, in times varying from nine to twenty-one days. All the fatal cases manifesting unusual cerebral symptoms occurred in private families, in which no post-mortem examinations could be obtained. Those coming under my own observation were in the south half of the West Division of the city; and it may be proper to remark that cases of cerebro-spinal meningitis were occurring with unusual frequency coincidently in the same part of the city.

With the exception of the class of cases I have just been describing, the general character of the symptoms in the pneumonic attacks of the past year in this city has been such as to indicate a decided typhoid or asthenic grade of morbid action. In only a few instances has the fever in the early stage exhibited such a degree of periodicity as to indicate the presence of a distinct malarious influence.

In regard to the treatment of pneumonia, I will detain you for only a few words concerning the more important items or questions that the subject suggests. The three principal sources of danger to life from acute pneumonic inflammation are, first, the extent and intensity of the vascular engorgement in the first stage of the inflammatory process.

When the disease attacks the greater part of both lungs simultaneously, constituting full double pneumonia, as it occasionally does, both in children and adults, the compression of the alveoli or air cells from the over-distension of the network of capillaries surrounding them, may diminish the amount of air received to such a degree as to prevent the oxygenation and decarbonization of the blood. The respirations become hurried, panting and unsteady; the pulse feeble and frequent, while the heart at first beats excitedly, but soon gives indications of weakness and unsteadiness; the mind, at first excited and anxious, soon becomes dull, and in some cases incoherent, while the whole external surface, including especially the face, neck, and trunk of the body, appears first congested, then mottled with purplish spots, and finally cyanosed, with cold extremities, entire collapse and death. Such cases, in which the fatal result is from apnœa, or the direct exclusion of air, are of rare occurrence, not more than five or six having come under my own observation in a period of forty-five years.

The second, and much more frequent source of danger to the life of the patient, is the amount of the exudation into the lung tissue and alveoli during the second stage in the progress of the disease. The exudation exerts a two-fold influence, namely, by depleting or actually diminishing the amount of blood in circulation, and by diminishing the oxygenation and decarbonization of the blood, from the exclusion of air from a large proportion of the alveoli of the inflamed part of the lung. With from one to three pounds of the elements of blood taken out of the circulation in the form of exudative material, and solidified in the alveoli and interstitial spaces of the lung structure, thereby excluding an equal bulk of air, you will readily see how a strong sedative or depressing effect is produced on the functions of both respiration and circulation, and why the cardiac force should be impaired, even to a dangerous degree, in the early part of the second stage.

The third source of danger is from the extent of purulent degeneration of the exudate, causing gray hepatization or diffuse suppuration instead of resolution, and progressive exhaustion of flesh and strength until death results from asthenia. It is thus evident that the cardiac weakness in the different forms, or rather stages, of pneumonia, which nearly all writers of the present time mention as the chief source of danger, and on which they found their use of particular remedies, is only a symptom or effect, resulting, in the first and second stages, from the sedative effect of imperfectly arterialized blood, and in the third stage chiefly from the extent of the suppurative process in the inflamed structures.

If these views concerning the actual pathological conditions that may endanger the life of the patient are correct, the objects most necessary to accomplish by treatment become obvious and well defined, namely, first, to limit the vascular fullness or accumulation of blood in the vessels of the inflamed part, and lessen the morbid excitability of the texture in the first stage; by which we shall prevent a dangerous degree of direct compression of the air cells in double pneumonia, and most efficiently limit the amount of exudation which is to constitute the chief source of danger in the second stage.

There are three practicable methods by which the quantity of blood in a part may be diminished. First, by abstracting a part of the blood, as by venesection, local bleeding, and other evacuants; second, by diminishing the force and frequency of the heart's action by cardiac sedatives; third, by increasing the tone or contraction of the smaller vessels of the part, through the agency of the vaso-motor nerves.

That a prompt, free bleeding in the first stages of active pneumonia is capable of lessening the fullness of the pulmonary vessels and relieving the pressure on the alveoli in a marked degree, I have demonstrated so many times as to have no possible doubt of its reality. It is equally true that such relief will, in a large proportion of the cases, prove temporary, if relied upon alone; but, if followed by the prompt and judicious use of such cardiac sedatives, coupled with mild anodynes, as will lessen the force and frequency of the cardiac action in the more sthenic cases, and by efficient doses of such remedies as promote an increase of the tone or contraction of the pulmonary vessels in the malarial and asthenic cases, the advantage gained by the bleeding will be perpetuated, thereby rendering the amount of exudation and red hepatization to constitute the second stage much less, and ensuring an earlier and more perfect recovery. It is true that in all the milder more limited cases of unilateral pneumonia the venesection may be dispensed with, even in the active or sthenic type of the disease. In such the cardiac sedatives during the first stage, accompanied and followed by a combination of anodyne and expectorant remedies, with rest and proper nursing is all the treatment required. But it is equally true that in the more severe cases of this type, the omission of the bleeding at the proper moment, greatly increases the danger of unfavorable progress, and has in times past been the occasion of many fatal results. When the pneumonic inflammation occurs in persons whose blood and tissues have been under the habitual influence of malaria, the effect of quinine in from five to ten grain doses, in restoring the tone of the pulmonary vessels and repressing the general febrile symptoms in the first stage, is in most cases prompt and efficient. I have seen some cases of this variety completely arrested within the first forty-eight hours after the initial chill, by taking five grains of sulphate of quinine with one of calomel and one of pulverized opium every three hours the first day and every six hours the second. After the latter a mild laxitive to move the bowels and three grains of quinine three times a day for four days was all the treatment required. In all ordinary cases occuring under malarious influences, the prompt and judicious use of quinine may take the place of blood-letting in the first stage of disease. But when the attack is severe, involving a large portion of one lung or portions of both lungs, and the patient comes under observation within twelve hours after the chill, a bleeding of from twelve to twenty ounces will render the action of efficient doses of quinine and opium more prompt and certainly beneficial than it would be without such loss of blood. When pneumonia occurs in the midst of sanitary conditions, favoring the prevalence of typhoid and typhus fevers, our reliance for diminishing the vascular engorgement of the first stage, must be mainly on the use of quinine, ergotine, and sponging the surface with cool water, or covering the whole chest with emollient poultices.

I have called attention thus fully to the treatment of the first stage of pneumonia, and the different agents that may be employed for accomplishing the same general object (relief of the vascular engorgement in the inflamed structure), and their adaptation to the treatment of cases occurring under different etiological conditions, because it is only by acting in this first stage promptly and judiciously, that we can materially limit the amount of exudation which is to follow and determine the danger or safety of the subsequent stages of each case. I had intended to allude to two or three other items of importance in the treatment of the second and third stages of the disease, but I have already occupied too much of your time, and will defer them until another opportunity offers.

